# Risk Stratification for Postoperative Mortality in Cardiac Surgery: “Quo Vadis”?

**DOI:** 10.3390/medicina62030606

**Published:** 2026-03-23

**Authors:** Radu-Alexandru Iacobescu, Tiberiu Lunguleac, Sabina Antoniu, Vlăduț Mirel Burduloi, Virgil Bulimar, Grigore Tinica

**Affiliations:** 1 Grigore T. Popa University of Medicine and Pharmacy Iasi, 700115 Iasi, Romania; radu_iacobescu@yahoo.com (R.-A.I.); sabina.antoniu@umfiasi.ro (S.A.); vburduloi@gmail.com (V.M.B.); vbulimar@hotmail.com (V.B.); grigoretinica@yahoo.com (G.T.); 2 Department of Thoracic Surgery, Respiratory Disease Hospital, 700115 Iasi, Romania; 3 Department of Respiratory Disease, Respiratory Disease Hospital, 700115 Iasi, Romania; 4 Department of Cardiovascular Surgery, Cardiovascular Disease Institute, 700503 Iasi, Romania

**Keywords:** cardiac surgery, surgical risk, surgery mortality, mortality risk, risk prediction modeling, machine learning

## Abstract

Risk assessment for immediate mortality is a vital component of the preoperative assessment in elective cardiac surgeries of the adult population. It is generally used to inform consent and plan postoperative care, but can also help identify patients who need preoperative optimization. Risk assessment for open cardiac interventions remains difficult, as an absolute risk assessment tool is still lacking. In this narrative review, we examine recent data on the predictive performance of commonly used risk assessment tools in cardiac surgery and explore missed opportunities to improve predictive performance, including overlooked independent predictors and alternative calculation strategies, such as machine learning. The literature shows that the most popular risk assessment tools are the Parsonnet score, EuroSCORE II, STS-PROM, and ACEF. These have reasonable discriminative capabilities across most populations but occasionally suffer from poor calibration and over- or underprediction. Preoperative inflammation, functional status, physical performance, nutrition, and frailty are potentially relevant clinical factors that could improve mortality prediction modeling using traditional approaches. By far, the largest advancement comes from artificial intelligence-based models that demonstrate superior predictive capabilities utilizing the same predictors. These models are still in development, have not received external validation, are not yet trusted by physicians, and may not be accessible to all institutions due to computing limitations, and thus are not ready for global rollout. Further research in identifying novel predictors of mortality is required, and efforts are needed to validate machine learning models in external cohorts.

## 1. Introduction

Safety in cardiac surgery has significantly increased over the past decades, attributable to improved surgical techniques and technology, as well as improved patient selection [1,2]. A decrease in the use of open procedures has been observed as a result of the development of minimally invasive techniques, yet in many instances, open surgery is unavoidable [3]. These advancements have enabled access to surgical treatments for an increasingly larger population of patients with more severe conditions [4,5]. In only a span of 10 years, the predicted operative risk of patients accepted for surgery has been reported to have increased by 30% [6]. Consequently, cardiac surgery in adults addresses a highly vulnerable population with increasing complexity, and continues to be considered among the most high-risk surgeries for undesired outcomes [4].

In-hospital mortality and 30-day mortality rates are perhaps not the best measurements of surgical safety; however, they are the most often reported concerns for open-heart surgery in adults [7,8,9]. Large cohort studies have indicated that mortality in cardiac surgery has steadily decreased from 4% to about 2.8% [10]. Expectably, mortality is higher for some cardiac procedures than others, previous reports showing rates of 2.3% for coronary artery bypass grafting (CABG) and 3.4% for valvular interventions [9]. More recent data show that mortality is roughly 2% for most interventions [8]. This is a non-negligible amount, considering the elevated number of patients evaluated for surgical intervention globally [5].

**Figure 1:**
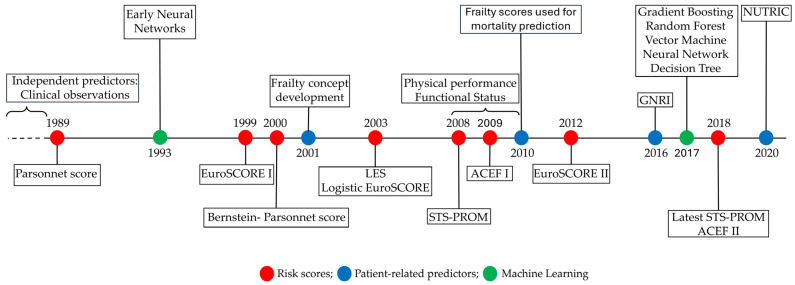
Timeline of risk assessment developments for mortality prediction in cardiac surgery.

Preventing undesirable outcomes, such as mortality, particularly in non-emergency settings, has been a focus of recent advances in risk assessment science [11]. Surgeons have been shown to have limited intuition in predicting surgical outcomes, regardless of experience [12,13]. Thus, other risk stratification strategies were required. Risk score-based stratification is a relatively recent development and remains subject to improvement. Several risk prediction models (scores) have been developed to predict mortality across different populations of cardiac surgical patients, most commonly reported being the Parsonnet Score, EuroSCORE (European System for Cardiac Operative Risk Evaluation) I and II, STS (Society of Thoracic Surgery) Score, and ACEF (Age, Creatinine and Ejection Fraction) [14,15]. These are mostly criticized for their internal validity constraints (such as overfitting and multicollinearity among predictors), their lack of calibration in populations other than those in which they were developed, their modest discriminative capability, and their failure to account for individual characteristics such as performance status and frailty [16]. Given this, the uptake of risk score utilization in clinical practice has been variable. New endeavors are now focused on building larger predictive models to improve accuracy using a machine learning (ML) approach [17]. This appears to be a more reasonable method for predicting mortality, but it remains under development, entails a significant delay before global rollout, and, to date, has limited availability. A timeline of these developments is presented in Figure 1.

Risk assessment is a critical component of preoperative evaluation that enhances decision-making and informs patient consent, yet it remains challenging in open cardiac surgery. In the current era, increasingly dominated by artificial intelligence, a comparison of predictive performance among independent predictors of mortality, ascertained predictive scores, patient-specific predictors, and machine learning models is warranted. In this article, we summarize data on the predictive performance of commonly used predictive scores for postoperative in-hospital mortality in adult patients undergoing open cardiac surgery, and compare results across different assessment approaches.

## 2. Independent Predictors of Postoperative Mortality in Cardiac Surgery

Before any modeling was considered, several empirical observations indicated that patients with specific characteristics tend to have worse outcomes than others [18]. In a retrospective on 11,190 cardiac surgeries from the STS (Society of Thoracic Surgery) databases, the most prevalent causes of postoperative death have been found to be cardiac (38.7%), renal failure 15.6%), and stroke (13.9%) [8]. Not surprisingly, age, creatinine levels, ejection fraction, stroke history, COPD, and infection were identified as independent predictors of mortality [19,20,21]. Consequently, these, or related biomarkers and predictors, are components of most existing predictive scores.

The search for other independent predictors continues. For instance, in a small sample study on surgical patients undergoing cardiopulmonary bypass (CPB), inflammatory biomarkers derived from complete blood cell count (CBC), such as NLR (Neutrophil-Lymphocyte Ratio), PLR (Platelet-Lymphocyte Ratio), and SII (Systemic Immune-Inflammation Index), were found to be predictors of in-hospital mortality along with gender, age, serum creatinine, hypertension, metabolism deregulation, and hemoglobin level [22]. In particular, SII was a prominent predictor in multivariate regression; however, the predictive power in ROC curve analysis remained limited for CBC-derived parameters (AUCs of 0.664, 0.655, and 0.690 for NLR, PLR, and SII, respectively; *p* < 0.001 for each). In a larger sample of CABG patients, Koyuncu et al. also show that NLR and PLR were predictors of mortality, with modest discriminative power [23]. The predictive value of the CBC-derived biomarker of inflammation appears limited, but could further improve the discrimination of established models.

Others considered serum biomarkers of inflammation, such as procalcitonin and C-reactive protein (CRP) [24]. A recent meta-analysis study of 29 studies researching the value of preoperative CRP as a predictor of mortality in cardiac surgery patients was conducted and found a risk increase of 1.88 (95% CI 1.60–2.20; I2 = 19%) for all-cause mortality, highlighting the importance of inflammation on postoperative outcomes and the role of serum biomarkers in predicting mortality [24]. Several studies have also considered procalcitonin (PCT) as a potential predictor. An early observation indicated that procalcitonin was a strong predictor of delayed adverse outcomes (AUC 0.9) in patients receiving CABG surgery [25]. Following, PCT was shown to be predictive of mortality in surgical patients in several prospective studies [26,27]. Still, in studies comparing PCT to already established scores, such as the EuroSCORE, it was found that this predictor was also insufficient [28]. Research on other serum biomarkers yielded similar findings. In a large multicenter study from Northern England, a biomarker panel comprising IL-6 and IL-10, along with ST2, galectin-3, N-terminal pro-brain natriuretic peptide (NT-proBNP), and cystatin C was assessed in a cohort of patients undergoing CABG and valvular surgery [29]. The predictivity of these was compared with that of established logistic composite models, such as the STS score. It showed that inflammation biomarkers were insufficient as standalone predictors of in-hospital mortality and other adverse outcomes, but may augment the predictive performance of existing scores (improving the AUC from 0.66 to 0.74 for the STS score). These findings are valuable for informing future predictive models that more comprehensively incorporate all relevant patient biological characteristics.

The search for unidentified predictors of mortality is commendable. These overlooked factors may represent a missed opportunity to improve mortality prediction. As an absolute model is still lacking, these predictors may provide an improved perspective on preoperative risk.

## 3. Score-Based Predictive Models from Development to Worldwide Validation

Score-based predictive models have emerged to integrate multiple independent predictors into a single risk estimate. Many additive and logistic regression risk prediction models exist; however, not all appear equally and ubiquitously effective. Selecting a risk assessment tool is generally at the surgeon’s discretion and is typically guided by the best available evidence; however, this remains challenging, as evidence shows variable reproducibility of results across different populations. Comparative studies have examined the predictive performance of these models across different cardiac surgery patient populations, revealing relatively uneven performance. A summary of findings from studies comparing discrimination of the most frequently reported risk scores is presented in Table 1.

### 3.1. Parsonnet Score

Among the first of its kind, the Parsonnet risk score was a first attempt to holistically stratify mortality risk in cardiac surgeries using a logistic regression approach [30]. Following its introduction in 1989, the inclusion of this assessment tool in the preoperative evaluation battery was met with immediate enthusiasm in many cardiovascular centers [31]. However, shortcomings immediately emerged, as observed and predicted mortality rates mismatched significantly [32]. A further revision and simplification of this score, under the name Bernstein-Parsonnet score, was found to be appropriately calibrated for patients undergoing CABG [33]. This revised version could better discriminate mortality than the EuroSCORE II in a surgical population in Brazil [34]. However, both these scores were developed early in the revolution of cardiac surgery, and safety has significantly improved since their initial development; consequently, data showed they usually overpredicted mortality and were not appropriate outside Western countries [16,35,36]. Most recent studies show this score underperforms in comparison to newer preoperative risk assessment tools [37,38].

### 3.2. European System for Cardiac Operative Risk Evaluation (EuroSCORE)

The first European-developed score to predict 30-day mortality was constructed in 1999 on a population of 14,781 patients from 128 surgical centers across eight European countries and used an additive risk approach (EuroSCORE I) [39,40]. A further logistic approach was applied to the dataset in 2003 to address the score’s tendency to underestimate risk in high-risk patients [41]. Although initially validated in several external surgical cohorts, the model calibration appeared to weaken over time, prompting reassessment of the included predictors and a readjustment of their weights in overall mortality prediction [42,43,44]. Consequently, in a larger endeavor comprising data from 154 centers from 43 countries and a robust cohort of 22,381 patients, a new variant of the additive model was proposed in 2012 (EuroSCORE II) [45]. This revised risk score showed reasonable applicability across several populations from Europe, Asia, South America, and Oceania, where it was most recently validated [37,44,46,47,48,49,50,51,52,53,54]. The discrimination power among the more recent studies is about 0.8. This is no different from a previous meta-analysis study comprising 22 relevant studies that was run two years following the score release, which found an estimated pooled AUC (area under the curve) of 0.792 (95% CI: 0.773–0.811) for postoperative mortality, which is considered to be good [55]. This suggests that the new score has corrected its initial time-dependent drift.

Still, the appropriateness of the EuroSCORE II across all surgical cohorts appears, so far, limited. This score has been shown to have poor calibration for several investigated populations (failed Hosmer-Lemeshow goodness-of-fit test with *p* < 0.05) [37,56,57,58,59,60,61,62,63]. Usually, it is criticized for underestimating mortality following surgery (observed to expected mortality rate-O/E > 1.00) [46,48,58,61,64,65,66,67]; however, in several instances, it has been found to overpredict it (O/E < 1.00) [37,58,59,60,68,69,70,71]. Despite efforts to include critical variables such as creatinine clearance and liver function, the underestimation of mortality persisted even in this updated variant, and the developers acknowledge the issue but deem it acceptable [45]. As this tool continues to receive global validation, several shortcomings are becoming apparent. Among them, the lack of consideration of patient frailty, liver dysfunction, advanced cardiac function metrics, and left ventricular dimensions are the main criticisms [72]. Since its re-evaluation, little effort has been made to resolve these issues, and with the rapid development of new tools underway, this previously well-established tool might soon become obsolete.

### 3.3. Society of Thoracic Surgeons Predicted Risk of Mortality (STS-PROM)

The first adult cardiac surgery database (ACSD) was pioneered in North America and endorsed by the Society of Thoracic Surgery (STS) in 1989 [73]. In 2011, it expanded to include international data from willing countries in order to increase data reliability [74]. It now includes over eight million cases from more than 3600 physicians globally [75]. The STS uses these data to constantly update and improve its own logistic predictive score for mortality in cardiac surgery (STS-PROM) [76,77].

**Table 1:** Comparison of the discriminative power of score-based prediction models.

Author [Ref.], Year, Study Design (Country), Study Population.	Cardiac Interventions	Prediction Scores	Discrimination for In-Hospital Mortality/30-Day Mortality AUC (95% CI)
Saka E. et al. [64], 2025, Prospective single-center (Turkey), *n* = 469.	CABG Valve surgery Combined	EuroSCORE II STS	0.783 (0.742–0.819) 0.823 (0.785–0.856)
Widyastuti Y. et al. [63], 2023, Prospective single-center (Indonesia), *n* = 1833.	CABG Other	EuroSCORE II ACEF	0.774 (0.714–0.834) 0.638 (0.561–0.718)
Koo S.K. et al. [65], 2022, Retrospective single-center (Australia) *n* = 898.	CABG Valve surgery Combined	EuroSCORE II STS AES LES AusSCORE ASMR	0.850 (0.78–0.92) 0.806 (0.71–0.90) 0.828 (0.74–0.92) 0.819 (0.73–0.91) 0.739 (0.62–0.86) 0.767 (0.66–0.87)
Boukhmis A. et al. [37], 2022, Prospective single-center (Algeria), *n* = 235.	CABG	EuroSCORE II STS Parsonnet	0.893 (0.798–0.987) 0.788 (0.617–0.959) 0.737 (0.520–0.953)
Santarpino G. et al., 2022 [69], Retrospective national database (Italy), *n* = 14,804.	CABG Valve surgery Combined Other	EuroSCORE II ACEF II	0.79 (0.79–0.80) 0.73 (0.73–0.74)
Shales S. et al. [58], 2021, Prospective single-center (India), *n* = 4895.	CABG	EuroSCORE II STS	0.71 (0.70–0.72) 0.72 (0.71–0.74)
Gao F. et al., 2021 [50], Retrospective single-center (China), *n* = 1628.	CABG	EuroSCORE II STS	0.900 (NA) 0.879 (NA)
Zhuo D.X. et al. [66], 2021, Retrospective single-center (USA), *n* = 259.	Valve surgery Combined	EuroSCORE II STS MAGGIC	0.688 (0.557–0.818) 0.797 (0.655–0.939) 0.721 (0.581–0.860)
Singh N. et al. [77], 2021, Retrospective single-center (New Zealand), *n* = 933.	CABG	STS AusSCORE II	0.921 (0.902–0.937) 0.882 (0.859–0.902)
Mejia O.A.V. et al. [52], 2020, Retrospective REPLICCAR registry (Brazil), *n* = 5222.	CABG Valve surgery Combined	EuroSCORE II STS SPScore	0.763 (NA) 0.766 (NA) 0.898 (NA)
Shapira-Daniels A. et al. [53], 2019, Retrospective single-center (Israel), *n* = 1279.	CABG Valve surgery Combined	EuroSCORE II LES STS	0.811(0.729–0.893) 0.810(0.735–0.902) 0.830(0.744–0.919)
Mejia O.A.V. et al. [34], 2018, Retrospective single-center (Brazil), *n* = 2919.	Valve surgery	EuroSCORE II Bernstein-Parsonnet RheSCORE InsCor Ambler score Guaragna score New York score	0.857 (NA) 0.876 (NA) 0.980 (NA) 0.835 (NA) 0.831 (NA) 0.816 (NA) 0.834 (NA)
Mateos-Pañero B. et al. [59], 2017, Prospective single-center (Spain) *n* = 866.	CABG Valve surgery Combined Other	EuroSCORE I EuroSCORE II SAPS III	0.862(0.812–0.912) 0.861(0.806–0.915) 0.692 (0.601–0.784)
Wang C. et al. [60], 2016, Retrospective multi-center (China), *n* = 12,429.	Valve surgery	EuroSCORE II STS New York score Ambler score	0.704 0.735 0.690 0.674
Ad N. et al. [68], 2016, Retrospective Single-center (USA), *n* = 11,788.	CABG Valve surgery Combined	EuroSCORE II EuroSCORE I STS	0.844 0.819 0.846
Chung W.J. et al. [38], 2015, Retrospective single-center (Taiwan), *n* = 305.	CABG	EuroSCORE I ACEF ACEF modified Parsonnet SYNTAX CSS Logistic CSS	0.75 (0.70–0.80) 0.76 (0.71–0.81) 0.73 (0.67–0.78) 0.68 (0.63–0.73) 0.54 (0.48–0.59) 0.72 (0.66–0.77) 0.76 (0.69–0.82)
Exarchopoulos T. et al. [54], 2015, Prospective single-center (Greece), *n* = 150.	CABG Valve surgery Combined Other	EuroSCORE II CASUS SOFA APACHE II SAPS II	0.87 (0.74–0.95) 0.89 (0.74–0.97) 0.76 (0.52–0.92) 0.82 (0.61–0.94) 0.80 (0.60–0.92)
Wang T.K.M. et al. [51], 2014, Retrospective single-center (New Zealand), *n* = 818.	CABG	EuroSCORE II LES STS AusSCORE I	0.642 (0.503–0.780) 0.675 (0.531–0.819) 0.641 (0.507–0.775) 0.661 (0.516–0.807)
Osnabrugge R.L. et al. [70], 2014, Retrospective national database (USA), *n* = 50,588.	CABG Valve surgery Combined	EuroSCORE II LES STS	0.77 (0.75–0.78) 0.78 (0.77–0.79) 0.81 (0.80–0.82)
Arnáiz-García M.E. et al. [61], 2014, Retrospective single-center (Spain), *n* = 1200.	CABG Valve surgery Combined Other	EuroSCORE II LES	0.745 (0.719–0.769) 0.758 (0.732–0.782)
Kirmani B.H. et al. [62], 2013, Retrospective single-center (UK), *n* = 15,497.	CABG Valve surgery Combined	EuroSCORE II STS	0.818 0.805
Borde D. et al. [71], 2013, Retrospective single-center (India), *n* = 498.	CABG Valve surgery Combined	EuroSCORE II STS	0.69 0.65
Qadir I et al. [44], 2012, Retrospective single-center (Pakistan), *n* = 380.	CABG	EuroSCORE I LES STS	0.866 0.842 0.899
Barili F. et al. [67], 2013, Retrospective multi-center (Italy), *n* = 1758.	Valve surgery	EuroSCORE I LES STS ACEF	0.81 (0.73–0.88) 0.80 (0.72–0.88) 0.85 (0.78–0.93) 0.78 (0.68–0.88)
Farrokhyar F. et al. [43], 2011, Retrospective national database (Canada), *n* = 3350.	CABG	EuroSCORE I STS	0.79 (0.71–0.88) 0.81 (0.73–0.90)

STS: Society of Thoracic Surgeons score, EuroSCORE: European System for Cardiac Operative Risk Evaluation, AES: Additive EuroSCORE, LES: logistic EuroSCORE; AusSCORE: Australian System for Cardiac Operative Risk Evaluation; ASMR: AusSCORE multi-risk, ACEF: Age, Creatinine and Ejection Fraction score; MAGGIC: Meta-Analysis Global Group in Chronic Heart Failure risk score; SAPS: European Simplified Acute Physiologic Scale; SYNTAX: Synergy between PCI with TAXUS and Cardiac Surgery score; CSS: Clinical SYNTAX Score; CASUS: cardiac surgery score; SOFA: Sequential Organ Failure Assessment; APACHE: Acute Physiology and Chronic Health Evaluation; CABG: Coronary Artery Bypass Graft.

The initial score was developed in 2008 from 774,881 CABG procedures, using ACSD data from 2002 to 2006, and had an initial discrimination of 81.2% [78]. This model was further validated for use in predicting mortality after other cardiac procedures [79,80]. The latest update of this model was released in 2018. It was constructed based on data from 1,250,165 CABG, valvular, and combined procedures dating from 2011 to 2016 (670,830 in the development cohort and 579,335 in the validation cohort) [81]. This model outperformed the previous one for all indexed operations [82]. The latest STS risk model incorporates up to 30 independent predictors, including demographics, serum biomarkers, and cardiac structural characteristics, which makes it perhaps the most robust risk assessment score available to date [16].

Not surprisingly, this score has received the largest support in practice, particularly in North America, but also from European circulating guidelines. Circulating guidelines of the ESC/EACTC (European Society of Cardiology and European Association of Cardio-Thoracic Surgery) on revascularization surgery appear to favor STS models at the expense of the EuroSCORE, as the former received stronger recommendations in 2018 [83]. This was, however, based on observations from a single study [70]. Emerging risk-stratification data prompted a reassessment of this recommendation, and, at least for CABG surgeries, it has been revised [84]. Still, recent studies show better calibration of the STS score than the EuroSCORE II [58,66,70]. However, it was also found to overpredict [37,64,65,70,71] or underpredict mortality in some populations [58,66].

### 3.4. Age, Creatinine, and Ejection Fraction Score (ACEF)

ACEF was developed to simplify risk assessment while maintaining discrimination in mortality prediction [85,86]. Its main advantage is that it reduces overfitting in populations with rarely observed events [63]. ACEF II was then developed in 2018 to include anemia and emergency of procedure as predictors to increase predictive power [87]. However, this later version proved no better than the previous one in later validation studies. In a Chinese surgical patient cohort of 9748 participants, no significant difference in AUC for mortality prediction was observed between the new and the previous version (AUC 0.704, 95% CI: 0.648–0.759 for ACEF II versus AUC 0.709, 95% CI: 0.654–0.763 for ACEF I) [88]. In comparative studies using other commonly used scores, both predictors show limited predictive power [38,63,67,69].

Since the development of these predictive scores, many external validation studies have been performed. Some studies found one score more appropriate for their population than others. However, there is a limited difference in predictability between the three. A meta-analysis study from 2016, considering 22 studies that compared the predictive power for mortality of ACFE, EuroSCORE, and STS, found that the pooled reported difference is insignificant or at least minimal [15]. Given this, the use of additive or logistic models for risk stratification to predict mortality has probably achieved its full potential.

## 4. Patient-Related Predictors, a Missed Opportunity

Some independent predictors used by the scores described in this article have, at least partially, lost their significance. Advanced age has been shown to be safe for well-selected elective heart procedures, and selecting patients for surgery solely on the basis of age is not recommended [89]. Moreover, the scores’ performance in mortality prediction was found to vary across age groups. For instance, in a small sample study on patients aged between 80 and 90 years, it was found that ACEF, EuroSCORE II, and Parsonnet score have limited predictability for one-year mortality [90]. In another sample of elderly patients undergoing cardiac surgery, Garzón-Rodríguez et al. found that EuroSCORE II is more suitable for predicting in-hospital mortality prediction within the octogenarian cohort compared to 65–79 age group (AUC-0.831; 95% CI: 0.6964–0.9656 versus AUC 0.6432; 95% CI: 0.5825–0.8095), while STS performed better in the 65–79 age group than in the group above 80 years (AUC 0.6971, 95% CI: 0.6042–0.7921 versus AUC 0.6894, 95% CI: 0.4931–0.8857) [91]. This is because in surgical patients, age’s contribution to surgical risk is likely multifactorial. Consequently, a dissection of these factors is underway, and functional status, physical performance, nutrition, and frailty are emerging as more coherent risk factors associated with aging (Table 2).

Physical performance has been identified as an important risk factor for undesirable outcomes following cardiac surgery. Gait speed and muscle strength have been correlated with postoperative in-hospital mortality in several elderly cohorts [92,93]. In a meta-analysis of 22 studies that assessed physical performance using the 6MD test (six-minute walk distance), it was found that low scores were highly predictive of postoperative mortality (HR 2.04, 95% CI: 1.48–2.83; *p* < 0.001) [94]. In a more recent prospective comparative study, Santana et al. analyzed several physical performance tests and compared them to the EuroSCORE II [95]. The study showed that, in elderly patients, the handgrip strength test had better discrimination for in-hospital mortality than the EuroSCORE II (AUC 0.80 versus 0.77) and that combining it with the EuroSCORE II improved the model’s discrimination to AUC 0.83, although this did not reach statistical significance (*p* = 0.18). Another study used the Short Physical Performance Battery (SPPB), a performance test that analyzes lower extremity function, to determine its predictability for unfavorable outcomes, including postoperative mortality, against the STS-PROM score [96]. It found that performance assessment can improve discrimination for STS in low-risk patients from 0.645 to 0.747 when combined. Taken together, this shows that improving model performance may rely on determining physical performance preoperatively.

Functional status assessment is also a critical component of the decision-making process in many hospital settings. Most elderly cardiac surgery patients exhibit altered functional status preoperatively, and cardiac surgery often further reduces functional capacity postoperatively [97,98]. However, the routine assessment of functional status in cardiac patients remains limited and typically involves a brief physical performance assessment without accounting for cognitive function [99]. Recent studies highlight the importance of including a comprehensive functional status assessment in the preoperative risk-prediction battery. In a large sample study, it was found that patients with low preoperative functional status, as assessed by the Barthel Index, had an increased risk of dying within 30 days of indexed surgery (HR 2.19, 95% CI, 1.35–3.53; *p* = 0.001) [100]. Another study on CABG patients found that altered functional status prior to surgery is highly correlated with mortality within 180 days following the procedure [101]. Still, data on the use of functional status assessment and its capacity to improve the predictive capabilities of ascertained models remain limited. Relevant data focusing on ADL scores (activities of daily living) are reported mainly for minimally invasive procedures [102]. New studies are needed to explore the use of this assessment to improve predictive model performance in open cardiac surgery.

Malnutrition is another component of aging-associated conditions, highly prevalent among cardiac surgery patients, that has a significant impact on postoperative outcomes and survival [103,104]. While STS and Parsonnet scores consider nutrition as a factor through BMI (Body Mass Index), other scores do not. Basing nutritional assessment solely on BMI is probably insufficient to determine its impact on postoperative outcomes. This is why several scores have been proposed for measuring nutrition [105]. Recent studies report on the use of the Geriatric Nutrition Risk Index (GNRI) and NUTRIC scores to predict postoperative mortality. GNRI has been shown to be a better discriminator of early mortality than BMI and albumin levels considered independently [106]. This score has been validated across several cardiac surgery populations. In a meta-analysis of 16 studies, it was shown that a low GNRI score is associated with an increase in short-term mortality (RR: 3.19, 95% CI: 1.68–6.07, *p* < 0.001; I^2^ = 39%) and long-term risk of death (RR: 2.32, 95% CI: 1.63–3.30, *p* < 0.001; I^2^ = 77%) [107]. In a comparative study, such as the one performed retrospectively by Boehm et al. on 6712 patients, it was found that GNRI had comparative discrimination to EuroSCORE II (AUC =  0.75 vs. AUC  =  0.71, *p*  = 0.051) while the combined model did not improve significantly (AUC  =  0.77, *p* = 0.6 for the observed difference) [108]. NUTRIC score serves as both a nutrition status assessment tool and a disease severity assessment instrument, and is recommended for use in critically ill patients [109,110]. A recent study that analyzed both NURTRIC and the modified version (mNUTRIC) potential for predicting in-hospital mortality in a cohort of 252 CABG patients found that these have a very good discriminating power (AUC 0.83, 95% CI: 0.78–0.87, and AUC 082, 95% CI: 0.77–0.87, respectively) [111]. However, to date, no comparison with already existing scores exists.

Finally, frailty emerged as an aging-associated multidimensional syndrome defined by a state of vulnerability to external stressors resulting from diminished physiological reserves [112]. It best describes altered functional status in patients with chronic conditions [113]. However, the difficulty lies in its assessment, as several definitions along with their measurement strategies have been proposed. Arguably, the most inclusive approach is the comprehensive geriatric assessment (CGA), which takes into account all the patient-related factors previously discussed, but it proves difficult to undertake in surgical settings [114]. This led to the development of several simplified frailty measurement instruments that can aid in stratifying risk preoperatively [102]. Regardless, frailty is an important risk factor for negative outcomes in cardiac surgery patients. A large meta-analysis study of 19 observational studies comprising 66,448 cardiac surgery patients found that frail and pre-frail individuals had a two-fold increase in in-hospital mortality risk (RR 2.35; 95% CI: 1.57–3.51; *p* < 0.0001 and RR 2.03; 95% CI: 1.52–2.70; *p* < 0.00001 for frail and pre-frail, respectively) [115]. In comparative studies, frailty assessment has been shown to improve the performance of other predictive scores. For instance, a study of 640 surgical patients aged 75 or older that used the Edmonton Frailty Score (EFS) to determine frailty found that this score had fair discrimination for in-hospital mortality (AUC 0.69, 95% CI: 0.56–0.82), but increased the discriminating power of EuroSCORE II by 3% (*p* = 0.04) [116]. A study that used Comprehensive Assessment of Frailty (CAF) and its simplified variant (FORECAST) to investigate frailty predictability for negative outcomes and in-hospital mortality and compared it to the EuroSCORE II and STS models found that frailty scores had similar discrimination capability (AUC 0.7, 0.69, 0.68, 0.64 for STS, CAF, FORECAST, and EuroSCORE II respectively) and that frailty increased the discrimination of the previous models, but the difference was not statistically significant (STS + CAF AUC 0.73, a 3% increase *p* = 0.27, STS + FORECAST AUC 0.72, a 2% increase *p* = 0.43, EuroSCORE II + CAF and EuroSCORE + FORECAST AUC 0.69, a 5% increase with *p* = 0.15 and *p* = 0.14 each) [117]. Another recent study used mFI-11 (modified frailty index with 11 items) to assess postoperative risk and found similar results (AUC 0.73, 95% CI: 0.62–0.85 for mFI-11, AUC 0.79, 95% CI: 0.68–0.90 for EuroSCORE II, and AUC 0.78, 95% CI: 0.67–0.89, when combined, respectively) [118]. Thus, frailty assessment preoperatively might represent a solution to increasing the predictive accuracy of already existing scores in a more comprehensive manner.

Unlike age, which is not modifiable, these aging-related conditions are potential targets for targeted interventions that may improve patients’ preoperative condition and increase their resilience to surgical stress [119]. These factors should not be treated individually but addressed through intervention together through a bundle of care [120]. Consequently, accurate determination of physical performance, functional status, nutrition, and frailty in the preoperative setting is paramount for constructing a tailored prehabilitation plan that can reduce negative postoperative outcomes.

## 5. Machine Learning and Artificial Intelligence in Predicting Postoperative Mortality: A Novel Approach

Artificial intelligence (AI) is a recent technological breakthrough that has revolutionized medical data analysis [121,122]. In surgical settings, it allowed for a larger amount of clinical and paraclinical data, including patient-related characteristics, to be utilized for a single prediction estimate [123]. The AI-driven algorithm approach has been used to increase diagnostic accuracy, improve image analysis, forecast health outcomes, and identify at-risk groups, thereby supporting preventive strategies, the development of personalized care, and healthcare delivery [124]. In cardiac surgery, machine learning models have significantly advanced preoperative risk assessment precision and improved patient outcomes [125]. Given this, extensive research has been carried out to develop novel predictive models for cardiac surgical outcomes using this approach, and a surge of AI-driven prediction models has been noticed since 2017 [126].

Data on the performance of existing AI models in cardiac surgery have recently been summarized in a scoping review by Khodaveisi et al. [124]. The analysis included 64 studies, of which 26 investigated ML models predicting postoperative mortality. It found that XGBoost and random forest modeling approaches were superior to logistic regression modeling in most studies, becoming preferred approaches in recent years for risk assessment [124]. The predictive performance of these models is unprecedented in cardiac surgery, with one study reporting that an XGBoost model achieved a discrimination AUC of 0.94 for severe negative outcomes, including death [127]. A meta-analysis study of 15 studies with a sample size of 134,230 cases found a pooled discrimination of 0.88 (95% CI: 0.83–0.93) for machine learning models in mortality prediction, which was higher than the pooled logistic regression approach reported, which was 0.81 (95% CI: 0.77–0.85), *p* = 0.03 for the difference [17]. Similar results were found in another meta-analysis by Penny-Dimri et al., which included 51 studies [128]. They identified a pooled discrimination of 0.81 (95% CI: 0.78–0.84) for ML models, while logistic regression displayed a pooled AUC of 0.79 (95% CI: 0.73–0.84) for in-hospital mortality. Comparative studies that also used conventional risk assessment scores show that this approach surpasses existing scores in both accuracy and reliability. In a large retrospective analysis of 168,565 surgical patients from 105 reporting centers in China, all ML models assessed were superior to EuroSCORE II and SinoSCORE II (additive, logistic, or refitted), with the best-performing approaches being logistic ML and XGBoost (*p* < 0.001) [129]. Another large sample study of 227,087 cases from the UK database used a machine learning approach using variables of EuroSCORE and found that XGBoost and random forest (RF) were the best performing models, although all ML models outperformed EuroSCORE II in both the training and validation datasets (AUC 0.834, 95% CI: 0.834–0.834 for XGBoost, AUC 0.834, 95% CI: 0.833–0.834 for R, and AUC 0.818, 95% CI: 0.817–0.818 for EuroSCORE II in the validation cohort, respectively) [130]. These studies show that these novel ML methods have improved the predictive performance for negative outcomes in cardiac surgery beyond what was previously possible with conventional logistic regression.

These models offer several benefits beyond performance. One is that they can be integrated within patient data-management software, and the needed risk assessment guidance can be provided without the need for additional data feed [131]. Additionally, these could provide real-time clinical decision support, as prediction could be updated as the routine clinical workflow progresses. This might also help identify dynamic clinical risk factors that are difficult to spot using conventional research designs. AI is thus a promising tool for individualised care and treatment optimisation, surpassing what is currently possible through conventional risk management.

Despite the overwhelming amount of data supporting the superiority of ML modeling, these face several shortcomings and barriers that hinder widespread clinical implementation. From a development standpoint, most of these models are based on retrospective single-center data and lack external validation. There are significant concerns about dataset bias in the development stage of AI-driven models [132]. Most ML models are developed on limited datasets that may not adequately represent the diversity of the surgical populations, healthcare infrastructures, and clinical practices. This limits their generalizability, a fact acknowledged in many studies [133]. Thus, large-scale prospective multicenter studies are needed to comprehensively validate the superiority of this novel approach. However, this appears challenging. ML models are proprietary algorithms not yet publicly available, unlike existing scores, which are easily accessible as online risk calculators. Also, integrating these risk calculators into the existing electronic record infrastructure might present significant barriers, including the need for additional computational infrastructure and standardized data collection. Not all clinicians have the infrastructure to support the computing resources needed for these algorithms to run effectively, which will likely delay global access. Another concern is that real-world clinical data contain unavoidable noise due to missing data, coding inconsistencies, and variable documentation, which could impact model reliability in an actual clinical environment [134]. ML models’ performance has been shown to temporally drift in a similar magnitude to logistic regression models in cardiac surgery, making them dependent on frequent recalibration and necessitating continuous monitoring of performance [135]. Ethical concerns have also emerged, pointing to data privacy and the implications for patient autonomy and accountability for potential misperformance [136]. Uptake is also reportedly limited by the lack of transparency into how these predictions are arrived at (the black-box effect), which limits clinician trust [124,133]. In this regard, promising progress has been made with the use of Explainable Artificial Intelligence (XAI), which is said to bridge the gap between software and clinical use, empowering surgeons to interpret, validate, and trust AI-derived predictions [137,138]. On the other hand, automation bias has been described as the risk of over-reliance on artificial intelligence applications and the inadvertent overlooking of contradictory clinical evidence [139]. Thus, AI-driven data should be carefully considered in the decision-making process by both patients and providers, and a degree of scepticism should always be maintained. Despite this, machine learning is perhaps the most reasonable approach to help guide risk assessment preoperatively in the future.

Modeling can further be improved even through AI. This could probably be achieved by expanding the data sources included. A comprehensive model to include clinical data, medical imaging, patient-related predictors, and real-time physiologic information is not available. This multimodal approach might further improve accuracy. However, further research is required. Ultimately, randomised clinical trials are needed to provide evidence that these models translate into measurable improvements in patient outcomes.

## 6. Conclusions

Risk assessment for postoperative mortality in cardiac surgery using traditional score-based models appears to have reached a limit of predictive performance. These scores display variable calibration across different populations, modest discriminative power, and usually do not account for patient-specific factors such as functional status, nutrition, and frailty. Predictive models could be further improved by integrating additional independent predictors or patient-specific factors, but this may increase the risk of overfitting. However, novel machine-learning approaches emerged as a more suitable way to determine surgical risk accurately. However, before global rollout, challenges related to external validation, interpretability, and accessibility must be addressed.

## Figures and Tables

**Table 2:** Emerging patient-related predictors.

Factor	Assessment Tool
Physical performance	6MD Test, Gait speed, Hand grip strength test
Functional status	Barthel Index, ADL
Nutrition	GNRI, NUTRIC
Frailty	CGA, EFS, CAF, mFI-11

## Data Availability

No new data were created or analyzed in this study.

## Abbreviations

The following abbreviations are used in this manuscript:

CABG	Coronary Artery Bypass Grafting
COPD	Chronic Obstructive Pulmonary Disease
EuroSCORE	European System for Cardiac Operative Risk Evaluation
STS	Society of Thoracic Surgery
ACEF	Age, Creatinine, and Ejection Fraction
AI	Artificial intelligence
ML	Machine Learning
CPB	CardioPulmonary Bypass
CBC	Complete Blood Cell Count
NLR	Neutrophil-Lymphocyte Ratio
PLR	Platelet-Lymphocyte Ratio
SII	Systemic Immune-Inflammation Index
CRP	C-reactive protein
6MD	Six-minute walk distance
SPPB	Short Physical Performance Battery
ADL	Activities of Daily Living
PCT	Procalcitonin
GNRI	Geriatric Nutrition Risk Index
CGA	Comprehensive Geriatric Assessment
EFS	Edmonton Frailty Score
CAF	Comprehensive Assessment of Frailty
AUC	Area Under the Curve
OR	Odds Ratio
HR	Hazard Ratio
RR	Relative Risk
RF	Random Forest
ESC/EACTC	European Society of Cardiology and European Association of Cardio-Thoracic Surgery
